# Broca’s area, responsible for speech production, is regulated by lung function

**DOI:** 10.4103/NRR.NRR-D-25-00685

**Published:** 2026-01-02

**Authors:** Siyu Cao, Wenwen Zhuang, Yuqian Hu, Shijun Qiu, Li-Hai Tan

**Affiliations:** 1 Department of Respiratory and Critical Care Medicine, Peking University Third Hospital, Beijing, China; 2 Guangdong-Hongkong-Macau Institute of CNS Regeneration and Key Laboratory of CNS Regeneration (Ministry of Education), Jinan University, Shenzhen Campus, Shenzhen, Guangdong Province, China; 3 Center for Language and Brain, Shenzhen Institute of Neuroscience, Shenzhen, Guangdong Province, China; 4 Department of Radiology, First Affiliated Hospital of Guangzhou University of Chinese Medicine, Guangzhou, Guangdong Province, China; 5 Guangdong Innovation Platform of Translational Research for Cerebrovascular Diseases, Shenzhen, Guangdong Province, China; 6 University International College, Macau University of Science and Technology, Macao Special Administrative Region, China

**Keywords:** Broca’s area, chronic obstructive pulmonary disease, dyspnea, functional magnetic resonance imaging, lung-brain axis, speech production, lung functions, microstructural changes, quantitative magnetic resonance imaging

## Abstract

For more than 150 years, Broca’s area—specifically, the pars opercularis and pars triangularis in the left inferior frontal gyrus—has been recognized as crucial for human speech production. However, it remains unknown why this region is recruited for speaking. Speech production involves not only conceptualization and motor planning but also respiration to provide the necessary airflow for creating sounds. Thus, the role of Broca’s area in speech may be shaped by the functionality and related brain regions of the lungs. To test this hypothesis, we recruited patients with chronic obstructive pulmonary disease and asked them to read words aloud while their brains were scanned using functional magnetic resonance imaging, with quantitative magnetic resonance imaging acquired separately. The chronic obstructive pulmonary disease patients exhibited altered cortical responses in the left inferior prefrontal cortex and other regions during speech tasks, and also had abnormal activation in cortical sites associated with breathing. In addition, using quantitative magnetic resonance imaging to generate longitudinal relaxation time (T_1_) maps as an index of brain microstructural changes, including dendritic maturation and myelination, we observed significantly longer longitudinal relaxation times in Broca’s area in the chronic obstructive pulmonary disease group than in the control group, suggesting reduced myelination and impaired microstructural integrity. Crucially, our data indicated that more severe dyspnea was associated with less well-developed microstructure in Broca’s area and weaker activation of this region. The present study indicates for the first time that the lungs may function to shape Broca’s area as the speaking center, providing novel evidence that pulmonary dysfunction can influence both the functional and structural properties of cortical language regions. These findings highlight the mechanistic role of the lung–brain axis in speech production and suggest potential targets for interventions to enhance speech performance.

## Graphical abstract

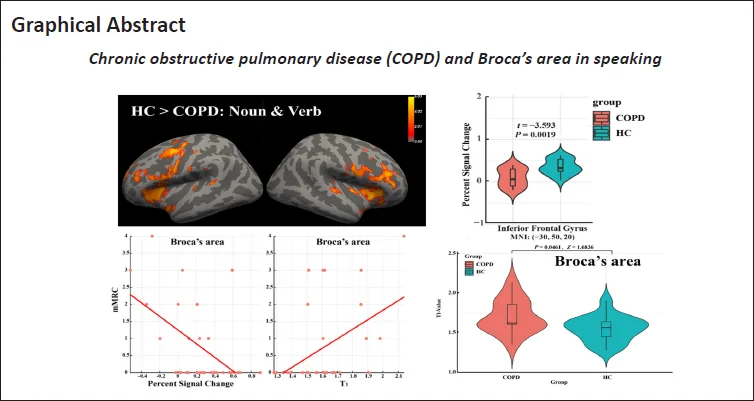

## Introduction

Broca’s area is located in the left inferior frontal gyrus; it is widely known as the speech production center, and was discovered by Paul Broca in 1861 (Broca, 1861). This brain area is located adjacent to the left precentral gyrus, and is assumed to be related to the motor planning and articulation stages of producing speech sounds (Ojemann and Mateer, 1979; Dronkers et al., 2007; Penfield and Roberts, 2014; Fedorenko and Blank, 2020; Duffau, 2021; Andrews et al., 2023; Chen et al., 2023; Hickok et al., 2023; Zhao et al., 2023; Fedorenko et al., 2024). In addition to coordinating speech-related motor movements, Broca’s area supports syntactic processing, semantic articulation, and the perception and production of sequential and hierarchical language structures (Wakita, 2020; Li et al., 2021; Maran et al., 2022). Lesions in this region typically lead to expressive deficits, such as Broca’s aphasia, thereby underscoring its importance for effective language output and communication (Lau et al., 2021; Agustina and Lestari, 2023). For example, Agustina and Lestari (2023) demonstrated that lesions in Broca’s area lead to phonological, morphological, and syntactic deficits, thus highlighting its critical role in language processing.

However, speech production is a complex process that involves not only conceptualization and motor planning, but also respiration, which provides the necessary airflow for speaking (Gilman et al., 2019; Boubenec, 2024; Park et al., 2024; Serré and Rochet-Capellan, 2024; Su et al., 2024). Air is drawn into the lungs and expelled through the trachea; this process is regulated by the brainstem and the bilateral inferior, middle frontal, and premotor cortices (Park et al., 2021; Ashhad et al., 2022), which facilitate sound production. Respiration is a fundamental neural rhythm that not only sustains vocal–respiratory coordination through brainstem circuits that control laryngeal motor activity (Park et al., 2024) but also entrains ongoing cortical activity via sensory pathways, thus linking higher-order cortical functions to respiratory processes (Heck et al., 2016). Given that sensory pathways are intricately connected to Broca’s area through a network of circuits that facilitate the processing of auditory and tactile information (Heine et al., 2015; Floegel et al., 2020), the functionality of the lungs may shape Broca’s area and related brain regions that enable effective language production and communication. Such respiratory–cortical coupling suggests that Broca’s area may be particularly vulnerable to pulmonary dysfunction. Because the operation of Broca’s area depends on tightly synchronized respiratory rhythms to support phonatory control and auditory–motor integration, we hypothesize that impaired lung function may disproportionately disrupt its role in coordinating fluent speech.

This hypothesis is in agreement with the recent animal model findings of a lung–brain axis, which indicate that the pulmonary microbiome may affect the central nervous system (Hosang et al., 2022). Recent advances in lung–brain axis research have demonstrated that pulmonary dysfunction can induce widespread effects on brain structure, connectivity, and cognitive performance (Guo et al., 2025; He et al., 2025; Huang et al., 2025). For example, Guo et al. (2025) performed bidirectional Mendelian randomization analyses on genome-wide data from 31,453 participants and revealed that several respiratory conditions, including chronic obstructive pulmonary disease (COPD), asthma, and severe COVID-19, exhibit causal effects on multiple brain networks (particularly, those governing attention and executive functions), thus providing genetic evidence for the lung–brain axis. Despite these insights, specific links to Broca’s area remain unexplored.

To test this hypothesis with human participants, we recruited a group of patients with COPD (**Additional Table 1**) as a clinically stable model of long-term respiratory impairment. Unlike transient or infectious pulmonary conditions, COPD enables the investigation of sustained lung–brain interactions. COPD exerts effects beyond pulmonary physiology, potentially affecting neural regeneration through chronic hypoxia and systemic inflammation (Martin et al., 2024; Mou et al., 2024). Hypoxia reduces oxygen availability, thereby affecting neural stem cell proliferation and differentiation (Dey et al., 2023), and systemic inflammation can disrupt the microenvironment required for neurogenesis and synaptic plasticity (Villalba et al., 2023). COPD may therefore alter the structure and function of Broca’s area as the result of respiratory–cortical coupling, thereby providing a mechanistic basis for the deficits in language production that are observed in patients with chronic lung impairment.

**Additional Table 1:** Demographic information of the participants

	COPD	HC	*t* value	*P* value
**fMRI**				
Age, yr	60.50 ±3.39	58.75 ±3.44	1.45	0.16^a^
Male	16 (100.0)	16 (100.0)	-	-
Height, m	1.69 ±0.05	1.66 ±0.05	1.42	0.16^a^
Weight, kg	65.47 ± 12.29	68.62 ±8.69	-0.84	0.41^a^
Body mass index, kg/m^2^	22.97 ±3.80	24.87 ±2.47	-1.68	0.10^a^
mMRC	1.56 ± 1.26	0	4.95	**0.000027** ^a^
Montreal Cognitive Assessment	19.56 ± 4.23	19.25 ± 3.89	0.22	0.83^a^
Educational background			-	0.78^b^
Primary school	3 (18.8)	3 (18.8)	-	-
Middle school	7 (43.8)	7 (43.8)	-	-
High school	5 (31.3)	6 (37.5)	-	-
Bachelor's degree	1 (6.3)	0	-	-
**qMRI**				
Age, yr	61.00 ±3.44	58.50 ±3.56	1.95	0.06^a^
Male	14 (100.0)	16 (100.0)	-	-
Height, m	1.68 ±0.05	1.65 ±0.06	1.58	0.13^a^
Weight, kg	63.61 ± 12.28	66.74 ±8.62	-0.82	0.42^a^
Body mass index, kg/m^2^	22.37 ±3.68	24.45 ± 2.33	-1.88	0.07^a^
mMRC	1.64 ± 1.39	0	4.73	**0.000058** ^a^
Montreal Cognitive Assessment	19.57 ±3.13	19.06 ±3.82	0.40	0.70^a^
Educational background			-	0.63^b^
Primary school	4 (28.6)	3 (18.8)	-	-
Middle school	5 (35.7)	7 (43.8)	-	-
High school	4 (28.6)	6 (37.5)	-	-
Bachelor's degree	1 (7.1)	0	-	-

The values are presented as the mean ± standard deviation or *n* (%). ^a^Obtained by two-sample *t* test. ^b^Obtained by chi-square test. *n* =16 and 14, respectively for fMRI and qMRI in the COPD group, respectively. *n* =16 and 16 for fMRI and qMRI in the HC group, respectively. COPD: Chronic obstructive pulmonary disease; fMRI: functional magnetic resonance imaging; HC: healthy control; mMRC: Modified Medical Research Council; qMRI: quantitative magnetic resonance imaging.

Prior studies have associated COPD with changes in brain structure and function (Finnegan et al., 2021; Wang et al., 2023; He et al., 2025; Liang et al., 2025). For example, Finnegan et al. (2021) applied unsupervised machine learning to stratify 91 COPD patients into high and low symptom-burden groups based on four potential dyspnea-related factors and observed that activation of the anterior insula was significantly greater in the low-burden group. Moreover, Wang et al. (2023) conducted a systematic review of 20 multimodal neuroimaging studies and reported widespread structural and functional brain alterations in COPD patients, including in the frontal, temporal, and hippocampal regions, as well as abnormal intrinsic brain activity in the posterior cingulate cortex, precuneus, lingual gyrus, and anterior central gyrus. Nevertheless, the implications of these COPD-associated structural and functional brain alterations for language-specific neural mechanisms remain largely unexplored. Language processing and production rely on distributed neural circuits that integrate cortical and subcortical sites. Neural regeneration and cortical plasticity enable the adaptive remodeling of language-related circuits (Pasquini et al., 2022; Nieberlein et al., 2023), thereby supporting speech production under chronic pulmonary dysfunction. These processes, including synaptic remodeling, dendritic growth, and white matter reconfiguration (Xing and Bai, 2020; Han et al., 2024), provide a substrate for preserving language processing and production, despite subtle structural or functional alterations. Assessing both functional activation and myelination therefore provides a comprehensive view of plasticity-related changes in cortical language regions in COPD patients.

Respiratory-based interventions that enhance pulmonary function can modulate brain activity (Goheen et al., 2023) and improve speech ability (Bausek et al., 2019), thus highlighting a potential pathway whereby improved lung function promotes cortical plasticity to support language function. For example, Bausek et al. (2019) demonstrated in a 4-week pilot study that combining resistive respiratory muscle training with a standard home-based COPD management program concurrently improved phonatory control and expiratory capacity, providing evidence that strengthening pulmonary function can facilitate speech production. These findings suggest that targeted respiratory interventions not only improve lung function, but also enhance neural plasticity within speech-related networks, thus providing a promising avenue for alleviating speech impairment in chronic lung disease.

Given the integral role of respiration in speech production, COPD provides a unique clinical resource for examining how chronic lung dysfunction may modulate Broca’s area. In the present study, we asked patients with COPD to overtly name commonly used high-frequency words while we scanned their brain activity and structure using magnetic resonance imaging (MRI). To minimize respiratory burden and ensure data quality, we did not use full sentences as stimuli, which may increase respiratory demands. Instead, we selected isolated nouns and verbs as experimental materials that engage partially dissociable neural systems in English (Shapiro et al., 2006), although this occurs less distinctly in Chinese. We contrasted their cortical activation and myelination alternation with healthy control (HC) participants, particularly focusing on Broca’s area and related cortical regions (such as the middle frontal gyrus and precentral cortex) that govern breathing. COPD is a progressive disease that is characterized by chronic irreversible airflow limitations in the lungs (Kim et al., 2024); it leads to changes in breathing-associated brain structure and function (Esser et al., 2016; Finnegan et al., 2021; Hudson et al., 2025). We therefore hypothesized that COPD patients would exhibit altered cortical responses during naming performance, and abnormal activity in cortical regions related to breathing function when speaking. This would constitute a mechanism by which lung function may be crucial to speech function associated with Broca’s area.

Using the lung–brain axis hypothesis as a base, the current study aimed to investigate how chronic pulmonary dysfunction influences language-related neural circuits, with a particular focus on Broca’s area. By combining functional MRI (fMRI) and quantitative MRI (qMRI) in COPD patients during a word-naming task, we sought to clarify the neural alterations in cortical activation and myelination that are associated with long-term respiratory impairment. The findings from this study will help to provide a scientific basis for understanding the role of pulmonary function in human speech production and offer potential implications for clinical approaches to speech and language disorders.

## Methods

### Participants

Thirty-five native Chinese participants (35 males, aged 53–65 years) participated in this study, including 18 patients with COPD and 17 HCs. The COPD patients were referred by pulmonologists, whereas the HCs were recruited mainly through poster advertisements and referrals from acquaintances. All participants reported normal or corrected-to-normal vision that was sufficient for task performance. Recruitment and data collection were conducted from December 2023 to May 2024. Each participant completed a series of assessments, including a basic demographic questionnaire, the Modified Medical Research Council (mMRC) Dyspnea Scale (Perez et al., 2015; Athlin et al., 2021) to provide a comprehensive assessment of breathlessness severity, and the Montreal Cognitive Assessment (Sleight et al., 2023) to evaluate cognitive function. To ensure the validity of the study, all participants were carefully screened to exclude those with a history of substance abuse, other pulmonary diseases or neurological disorders, which might confound the results. Participants with COPD were included if they had a clinical diagnosis of mild to extremely severe COPD for over 1 year based on symptoms and previous pulmonary function tests, and had no other pulmonary diseases. The diagnostic criteria followed the Global Initiative for Chronic Obstructive Lung Disease guidelines (Agustí et al., 2023); namely, a post-bronchodilator ratio of forced expiratory volume in 1 second to forced vital capacity of < 70%. This study was approved by the ethics committee at the Shenzhen Institute of Neuroscience (Approval No. SION202305) on October 16, 2023, and all participants provided informed consent for their participation. The study was conducted in accordance with the Declaration of Helsinki. Detailed demographic information is provided in **Additional Table 1**.

### Stimuli and experimental design

The study included two experimental tasks: the Noun Chinese Character (e.g., “花” means “flower”) and the Verb Chinese Character (e.g., “踢” means “kick”) tasks. In both tasks, participants were instructed to overtly name the presented Chinese characters as quickly and accurately as possible. In the baseline condition, participants were required to maintain focus on the presented non-character symbols without a verbal response (**Additional Figure 1**). All Chinese characters were selected from a first-grade Chinese language textbook to ensure their familiarity among participants. A total of 48 nouns and 48 verbs were used, with 8 nouns/verbs allocated for practice and the remaining 40 nouns/verbs used in the formal experiment. The visual complexity of the stimuli was calculated using the number of strokes in Chinese characters (mean strokes: nouns = 9.23; verbs = 9.73). Baseline stimuli were generated by pairing and rotating common punctuation marks (e.g., “~&”) to match the upper/lower structure proportions of the characters to ensure that visual features were balanced across experimental conditions.

The experiment adopted a blocked design. The practice session consisted of three blocks (two experimental blocks and one baseline block). In the formal task, there were five blocks each for the Noun and Verb tasks and nine blocks for the baseline condition. Each experimental block contained eight trials, and each baseline block contained nine trials. During each trial, a stimulus (e.g., character or symbol) was displayed for 0.5 seconds, followed by a 2.5-second blank interval. The stimuli within each block were presented in pseudo-randomized order to minimize predictability and reduce potential biases. This design aimed to ensure a balanced and controlled presentation of stimuli across conditions.

### MRI data acquisition and preprocessing

T1-weighted structural MRI and fMRI data were collected on a Siemens 3-T MRI scanner (Siemens Healthcare, Erlangen, Germany) using a 64-channel head coil. High-resolution anatomical images were acquired using a T1-weighted, three-dimensional gradient-echo sequence with the following parameters: repetition time (TR) = 2300 ms, echo time (TE) = 2.07 ms, flip angle = 7°, voxels = 0.8 mm × 0.8 mm × 0.8 mm. The fMRI data were acquired using echo planar imaging sequences with the following parameters: TR = 2000 ms, TE = 30 ms, flip angle = 90°, voxel size = 3.5 mm × 3.5 mm × 3.5 mm, volumes = 211, field of view = 224 mm, acquisition matrix = 64 × 64, slice thickness = 3.5 mm. The visual stimuli were presented using a liquid crystal display projector and were back-projected onto a screen positioned at the rear of the scanner bore. Participants viewed this projection via a mirror attached to the 64-channel head coil. Prior to each functional scan, 6 seconds of dummy scans were conducted to facilitate the T1 equilibration.

Image preprocessing was performed using the SPM12 (http://www.fil.ion.ucl.ac.uk/spm/software/spm12) and DPARSF (http://rfmri.org/DPARSF) toolboxes. The T1-weighted images were segmented into white matter, gray matter, and cerebrospinal fluid using DARTEL implemented in DPARSF to avoid signal confusion among these three tissue types (Zhuang et al., 2023). For fMRI data, preprocessing steps included removing the first three images, slice timing correction, realignment, co-registering T1 images to the functional images, normalizing to Montreal Neurological Institute (MNI) space using DARTEL, resampling to 3 mm × 3 mm × 3 mm voxels, and applying 6-mm full width at half maximum Gaussian smoothing. To ensure data quality, participants with a maximum head motion > 3 mm or a maximum rotation > 3° were excluded from the study. All preprocessing and quality control steps were implemented in DPABI (https://rfmri.org/DPABI), which provides an integrated pipeline for motion correction and spatial normalization. After these procedures, 32 participants (32 males, 53–65 years) were selected (for detailed information, see Table S1) for further fMRI analyses.

**Table 1:** MNI coordinates and activation statistics of the regions

Hemisphere	Regions	Broca’s area	Number	MNI coordinates (mm)			*Z* score	Cohen’s *d*
				*x*	*y*	*z*		
Left	Precentral cortex	6	304	–45	0	45	4.29	1.26
Left	Middle frontal gyrus	9	13	–45	9	45	3.72	1.24
Left	Postcentral cortex	43	36	–63	0	24	4.16	1.31
Left	Inferior frontal gyrus	44	83	–51	9	27	3.86	1.24
Left		45	56	–45	39	21	4.1	1.26
Left	Peri-insular cortex	48	282	–42	15	–3	4.62	1.25
Right	Precentral cortex	6	94	42	6	57	3.63	1.19
Right	Middle frontal gyrus	9	5	45	15	54	3.44	1.15
Right	Postcentral cortex	43	33	60	–3	24	3.57	1.23
Right	Inferior frontal gyrus	44	90	48	27	33	3.56	1.18
Right		45	95	45	30	18	4.24	1.27
Right	Peri-insular cortex	48	281	39	18	0	4.62	1.24

MNI: Montreal Neurological Institute.

### Quantitative MRI data acquisition and preprocessing

The parameters of qMRI were determined according to the protocols of previous studies (Mezer et al., 2013; Yuan et al., 2021). The MRI data were acquired using a 3-T Siemens Magnetom Prisma scanner (Siemens Healthcare) equipped with a 64-channel head coil. Quantitative measurements of macromolecular tissue volume and longitudinal relaxation time (*T*_1_) were obtained from spoiled gradient echo images with flip angles of 4°, 10°, 20°, and 30° (TR = 12 ms, TE = 2.41 ms), at a voxel size of 1 mm × 1 mm × 1 mm. Additionally, five spin-echo inversion recovery images were scanned using an echo-planar imaging readout, a slab inversion pulse, and spectral fat suppression to correct for field inhomogeneities. The images were captured at inversion times of 50, 200, 400, 1200, and 2400 ms (TR = 3000 ms, TE = 49 ms), with a voxel size of 2 mm × 2 mm × 7 mm. Two participants were registered and resampled with other participants because of inconsistent slice thicknesses. Finally, 30 participants underwent follow-up qMRI analysis (for detailed information, see **Additional Table 1**).

### Data analysis

For individual-level analysis, all individual participant t-maps (i.e., Noun Chinese Character versus Baseline, Verb Chinese Characters versus Baseline, Noun Chinese Character versus Verb Chinese Characters, Verb Chinese Characters versus Noun Chinese Character, and Noun Chinese Character + Verb Chinese Characters versus Baseline) were generated using a general linear model based on smoothed data in which the time series were convolved with the canonical hemodynamic response function and high-pass-filtered at 128 seconds. Head motion parameters of six dimensions from realignment were modeled as regressors of no interest (Siok et al., 2020; Chao et al., 2021).

The contrast images were subsequently analyzed at the group level using a second-level model. To assess the effects of COPD on brain activation, a two-sample *t*-test was adopted to contrast brain activity between two groups. The significance threshold was set at *P* < 0.05 (voxel-wise false discovery rate [FDR] correction) (Siok et al., 2020) with a spatial extent ≥ 10 voxels (Luo et al., 2019). In addition, we applied two voxel-wise thresholds (*P* < 0.05 voxel-wise FDR-corrected and *P* < 0.001 uncorrected) and performed an analysis of the two groups of participants under the baseline condition.

To further validate our group-level findings, we adopted a region of interest (ROI) analysis using MNI coordinates that have been reported as relevant to lung function in prior studies, including the bilateral pars opercularis (MNI: −54, 8, 16; 50, 10, 18) (Evans et al., 2002), left inferior frontal triangularis (MNI: −50, 36, 22) (Chan et al., 2018), left inferior frontal gyrus (MNI: −30, 50, 20) (McKay et al., 2008), left middle frontal gyrus (MNI: −40, 40, 16) (Chan et al., 2018), right pre-supplementary motor area (MNI: 4, 12, 56) (Evans et al., 2002), left inferior precentral gyrus (MNI: −52, −8, 50) (McKay et al., 2003), left middle insula (MNI: −42, 2, 2) (McKay et al., 2008), and right insula (MNI: 38, 10, 8; 45, 16, −2) (Raux et al., 2013). For each literature-based coordinate, a spherical ROI with a 6-mm radius was created, and the percent signal change was calculated by normalizing contrast estimates to the implicit baseline using the MarsBaR toolbox (https://marsbar-toolbox.github.io/about.html). Between-group differences were then assessed using two-sample t-tests, with a significance threshold set at *P* < 0.05 FDR-corrected.

The spoiled gradient echo and spin-echo inversion recovery images were processed using the mrQ toolbox (https://github.com/mezera/mrQ) in MATLAB to generate participant-specific *T*_1_ maps (Yuan et al., 2021; Shao et al., 2022). *T*_1_ maps provide voxel-wise longitudinal relaxation times, adding in tissue properties such as myelination and fluid content. To focus on Broca’s area (left Brodmann area [BA] 44), a mask was extracted from the Brodmann atlas and aligned with the *T*_1_ map of each participant. The mask was resampled to ensure spatial consistency across participants, and mean *T*_1_ values for Broca’s area were calculated. A non-parametric rank sum test was used to compare *T*_1_ values between two groups. Additionally, the Cohen’s d values of mean *T*_1_ values were evaluated to quantify the effect size between two groups within Broca’s area. We further computed the partial correlations between mean *T*_1_ values and percent signal change values in the left BA 44, controlling for education levels.

Using the contrast image of the two groups of participants (HC > COPD) in the contrast condition (experimental versus baseline conditions), the total activated voxels/volume of each region were extracted by intersecting the activation map (FDR-corrected, *P* < 0.05, spatial extent ≥ 10 voxels) with the Brodmann map. To ensure comparability, the Brodmann mask from DPABI was resampled to match the spatial resolution of the activation maps. To quantify the effect size of the observed differences in brain activation between the COPD and HC groups within the identified regions, Cohen’s *d* was calculated for each region based on the *T*-values derived from the second-level group analysis. The equation for Cohen’s *d* based on *T*-values was as follows:


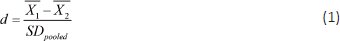


where 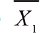 and 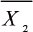 represent the mean activation values for the COPD and HC groups, respectively, and *SD*_pooled_ is the pooled standard deviation across the two groups. The equation for the independent samples *t*-test was as follows:


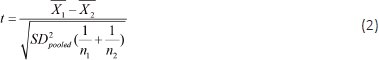


where *n*_1_ and *n*_2_ represent the sample sizes for the COPD and HC groups, respectively.

Combing equations (1) and (2), the equation that was used to calculate Cohen’s *d* from the *T*-value was as follows:


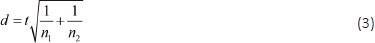


Finally, we calculated Pearson’s correlation coefficient to evaluate the relationship between mMRC dyspnea scores and percent signal changes, which were extracted from each brain site. We also analyzed the correlation between mean *T*_1_ values in the left BA 44 and mMRC scores. Both types of correlation analyses used a threshold of *P* < 0.05. Higher mMRC scores indicate more severe breathing symptoms.

## Results

### Atypical brain activation in chronic obstructive pulmonary disease patients

**Figure 1:**
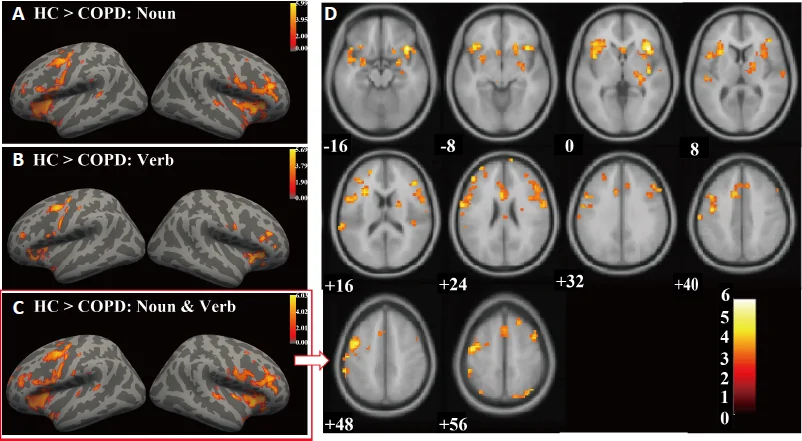
Statistical parametric maps of group activation differences between tasks (false discovery rate-corrected *P* < 0.05 with a spatial extent of *n* ≥ 10 voxels). (A) The group differences during the Noun contrasted with the non-character symbols. (B) The group differences when the verb is contrasted with the non-character symbols. (C, D) The group differences during the Noun and Verb conditions contrasted with the non-character symbols. COPD: Chronic obstructive pulmonary disease; HC: healthy control.

Because we asked our participants to read out nouns and verbs in Chinese, and then compared the results with a baseline condition in which the participants passively viewed non-word symbols, we first contrasted the brain activation of the two groups in each of the two experimental conditions (relative to the baseline). Activation was significantly weaker in COPD patients than in controls when naming nouns as well as verbs (**Figure 1A** and **B**, FDR-corrected, *P* < 0.05), and there was no clear difference in brain activation between nouns and verbs (**Additional Figure 2**), consistent with the findings of previous research (Li et al., 2004; Chan et al., 2008).

We then collapsed the data from nouns and verbs for further analyses. We focused on brain regions that are closely associated with naming. Activation in the COPD group was significantly weaker than that in the HC group (**Figure 1C**, **D**, and **Additional Table 2**) across multiple regions, including the bilateral precentral cortex (BA 6; MNI: −45, 0, 45; 42, 6, 57), bilateral postcentral cortex (BA 43; MNI: −63, 0, 24; 60, −3, 24), and bilateral peri-insular cortex (BA 48; MNI: −42, 15, −3; 39, 18, 0). Importantly, we identified weaker activity in several key speech production areas in the COPD group, including the bilateral inferior frontal gyrus (BA 44/45; MNI: −51, 9, 27; −45, 39, 21; 48, 27, 33; 45, 30, 18), bilateral middle frontal gyrus (BA 9; MNI: −45, 9, 45; 45, 15, 54), and bilateral precentral cortex (BA 6; MNI: −45, 0, 45; 42, 6, 57). The Cohen’s d values for these regions were all > 0.8, indicating a large effect size and suggesting a reliable difference in brain activation between the groups in these regions (**Table 1**). No significant differences were observed between the two groups of participants in the baseline condition. This null result indicates that the main findings reported in the study are not confounded by baseline activity, and thus are likely to be related to task-specific speech production.

**Additional Table 2:** MNI coordinates of the activation peaks

	BA	Left hemisphere						Right hemisphere					

		num	x	y	z	T score	Z score	num	x	y	z	T score	Z score
	1	8	-63	-21	39	4.05	3.59	^-^	^-^	^-^	^-^	^-^	^-^
Postcentral gyrus	2	11	-48	-39	60	3.71	3.34	^-^	^-^	^-^	^-^	^-^	^-^
	3	13	-42	-12	39	4.35	3.8	^-^	^-^	^-^	^-^	^-^	^-^
Primary motor cortex	4	47	-45	-9	39	4.43	3.85	1	54	-3	27	3.06	2.83
Precentral cortex	6	304	-45	0	45	5.10	4.29	94	42	6	57	4.10	3.63
Precuneus	7	8	-33	-72	54	3.70	3.33	26	36	-69	57	4.09	3.61
Superior frontal gyrus	8	8	-3	18	51	3.72	3.35	23	21	27	60	3.82	3.42
Middle frontal gyrus	9	13	-45	9	45	4.23	3.72	5	45	15	54	3.84	3.44
Frontopolar cortex	10	9	-30	60	18	4.70	4.03	11	12	69	24	4.15	3.66
Orbitofrontal cortex	11	-	^-^	^-^	^-^	^-^	^-^	24	18	21	-12	4.73	4.06
Inferior temporal gyrus	20	7	-39	-3	-21	3.55	3.22	24	30	-24	-3	4.03	3.58
Middle temporal gyrus	21	15	-42	3	-21	5.13	4.31	1	42	3	-21	3.01	2.79
Superior temporal gyrus	22	14	-66	-33	15	4.13	3.64	26	63	-18	6	3.95	3.52
Anterior cingulate cortex	24	40	-6	15	36	4.02	3.56	5	3	21	27	3.23	2.97
Subgenual cingulate cortex	25	12	-6	15	-9	3.76	3.37	11	0	18	0	4.11	3.63
Presubiculum	27	2	-6	-30	-6	3.55	3.22	^-^	^-^	^-^	^-^	^-^	^-^
Subdivision of retrosplenial cortex	30	10	-12	-39	-21	4.42	3.85	^-^	^-^	^-^	^-^	^-^	
Anterior cingulate cortex	32	62	-12	12	39	4.22	3.71	40	9	30	36	4.03	3.57
Dorsal entorhinal cortex	34	20	-24	0	-12	4.05	3.58	27	27	0	-15	4.05	3.59
Medial temporal lobe	36	4	-39	0	-21	5.12	4.3	^-^	^-^	^-^	^-^	^-^	^-^
Fusiform gyrus	37	^-^	^-^	^-^	^-^	^-^	^-^	15	30	-27	0	3.64	3.29
Anterior temporal pole	38	59	-39	3	-21	6.02	4.84	63	39	21	-15	5.56	4.57
Supramarginal gyrus	40	23	-54	-45	54	4.26	3.74	^-^	^-^	^-^	^-^	^-^	^-^
Auditory cortex	41	-	^-^	^-^	^-^	^-^	^-^	2	42	-30	12	3.28	3.00
	42	19	-63	-33	15	3.89	3.47	^-^	^-^	^-^	^-^	^-^	^-^
Postcentral cortex	43	36	-63	0	24	4.89	4.16	33	60	-3	24	4.03	3.57
Inferior frontal gyrus	44	83	-51	9	27	4.43	3.86	90	48	27	33	4.01	3.56
	45	56	-45	39	21	4.79	4.1	95	45	30	18	5.01	4.24
Middle frontal gyrus	46	67	-30	57	21	4.40	3.83	23	33	45	30	4.00	3.55
Orbitofrontal cortex	47	135	-39	21	-6	5.13	4.31	102	39	21	-3	6.03	4.84
Peri-insular cortex	48	282	-42	15	-3	5.64	4.62	281	39	18	0	5.69	4.65

BA: Broca’s area.

We next analyzed the correlation between mMRC dyspnea scores and the percent signal changes that were extracted from these brain sites. Our analysis revealed negative correlations between the percent signal changes extracted from these regions and mMRC scores (**Table 2**), particularly in the left inferior frontal gyrus (BA 44: *r* = −0.54, *P* = 0.0014; BA 45: *r* = −0.43, *P* = 0.015) and left peri-insular cortex (*r* = −0.56, *P* = 0.00085).

**Table 2:** Correlations between the mMRC Dyspnea Questionnaire scores and percent signal change in regions, as well as the mean *T*_1_ values in the left BA 44

	Regions	Peak MNI coordinates (mm)			mMRC *r* value
		*x*	*y*	*z*	
Percent signal change	Precentral cortex	–45	0	45	–0.58***
		42	6	57	–0.51**
	Middle frontal gyrus	–45	9	45	–0.45**
		45	15	54	–0.44*
	Postcentral cortex	–63	0	24	–0.54**
		60	–3	24	–0.49**
	Inferior frontal gyrus (BA 44)	–51	9	27	–0.54**
		48	27	33	–0.49**
	Inferior frontal gyrus (BA 45)	–45	39	21	–0.43*
		45	30	18	–0.32
	Peri-insular cortex	–42	15	–3	–0.56***
		39	18	0	–0.48**
mean *T*_1_ value	Inferior frontal gyrus (BA 44)	–	–	–	0.45*

*P < 0.05, **P < 0.01, ***P < 0.001. BA: Broca’s area; mMRC: modified Medical Research Council; MNI: Montreal Neurological Institute.

Speech production as a complex process is mediated by Broca’s area as well as regions that are responsible for articulatory gestures and auditory perception; these work together to coordinate the act of speaking and listening to ensure accurate speech output (Flinker et al., 2015; Leonard et al., 2023). Consistent with this theory, we identified weaker activity in the superior temporal gyrus (MNI: −66, −33, 15; 63, −18, 6) in COPD patients than in controls. Beyond these language-related regions, we also observed lower activation in the hippocampus (MNI: 31, −15, −15) in COPD patients than in controls.

COPD patients may have multiple neurocognitive impairments that are not specific to speech production. To address this issue, we analyzed brain activity provoked by the baseline condition, in which the two groups of participants passively viewed non-words. We observed no significant differences between the two groups (FDR-corrected, *P* < 0.05; uncorrected, *P* < 0.001). The Montreal Cognitive Assessment also indicated no significant differences between the two groups (COPD: 19.56 ± 4.23; HC: 19.25 ± 3.89; *t* = 0.22, *P* = 0.83; **Additional Table 1**). These results suggest that our findings may be specifically associated with speaking.

### Region of interests for breathing based on the literature

Because the middle frontal gyrus, pre-supplementary motor area, inferior frontal gyrus, precentral gyrus, and peri-insular area have been repeatedly demonstrated to be associated with breathing, we selected ROIs consistent with those reported in the published studies related to the lung function. We then analyzed these data to examine whether there are regional differences in brain activation between HC and COPD participants. The ROIs included the bilateral pars opercularis, left triangularis part of the inferior frontal gyrus, left inferior frontal gyrus, left middle frontal gyrus, right pre-supplementary motor area, left inferior precentral gyrus, left middle insula, and right insula.

**Figure 2:**
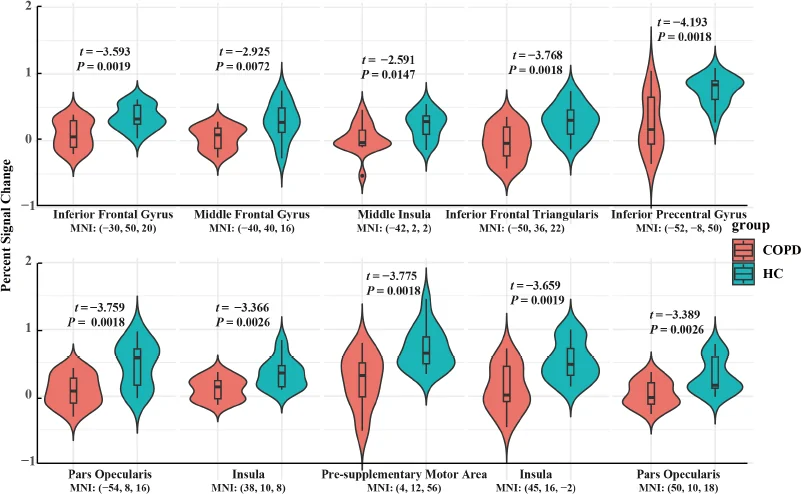
Activation statistics of the literature-defined regions. Each plot corresponds to a 6 mm-radius spherical region of interest centered at the peak activation coordinates reported in previous studies (MNI space). Percent signal changes were extracted from individual-level analyses comparing noun and verb conditions against baseline. The group differences were tested using two-sample *t*-tests. *P* values were corrected for multiple comparisons using the Benjamini‒Hochberg false discovery rate method. COPD: Chronic obstructive pulmonary disease; HC: healthy control.

Percent signal changes were extracted from the literature-defined ROIs for both groups, and a two-sample *t*-test was conducted. There was significantly higher activation in the HC group than in the COPD group (**Figure 2**). For example, the left pars opercularis (MNI: −54, 8, 16), left inferior frontal triangularis (MNI: −50, 36, 22), and left inferior frontal gyrus (MNI: −30, 50, 20) all showed a marked increase in activation in the HC group compared with the COPD group. Collectively, the data indicated that the neural function in lung-related regions of COPD patients was much weaker than that of HC participants, which may have further led to hypoactivation in naming.

### Microstructure differences in *T*_1_

**Figure 3:**
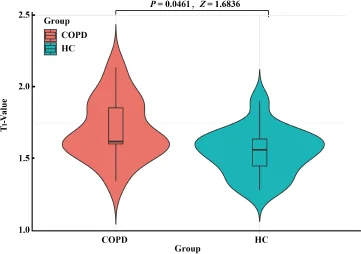
Statistical map of the mean *T*_1_ values in the left BA 44 comparing the COPD and HC groups using a right-tailed Wilcoxon rank-sum test. COPD: Chronic obstructive pulmonary disease; HC: healthy control.

To quantify cortical myelin architecture from a microstructural perspective, we used qMRI to generate *T*_1_ maps, which are linearly related to iron and myelin concentrations (Mezer et al., 2013; Stüber et al., 2014; Luo et al., 2019). A developmental decline in *T*_1_ is believed to reflect microstructural changes, such as dendritic maturation, myelination, and oligodendrocyte proliferation (Gomez et al., 2017). We calculated the average *T*_1_ values within Broca’s area (left BA 44) to assess group differences in tissue microstructure. The COPD group had significantly higher *T*_1_ values than the HC group (*Z* = 1.68, *P* = 0.0461; **Figure 3**). Cohen’s *d* for this region was 0.81, indicating a large effect size and suggesting a marked difference between the groups in Broca’s area. Given that higher *T*_1_ values are typically associated with lower tissue density and reduced myelination, these results suggest reduced myelination and microstructural integrity in the COPD group.

Pearson’s correlation coefficient revealed a significant positive correlation between mean *T*_1_ values in the left BA 44 and mMRC dyspnea scores (*r* = 0.45, *P* = 0.0130; **Table 2**). This finding suggests that higher *T*_1_ values are associated with greater mMRC scores, which reflect more severe dyspnea. However, we did not identify a significant partial correlation between the percent signal change and mean *T*_1_ values after controlling for education level.

## Discussion

In the present study, we investigated how pulmonary dysfunction in COPD modulates the neural substrates of speech production. Previous research into the lung–brain axis has primarily emphasized systemic mechanisms such as neuroinflammation, immune modulation, and microbiome-related processes. By contrast, the present work specifically targeted Broca’s area, which is crucial for speaking in humans. By integrating fMRI and qMRI techniques, we identified both functional and microstructural alterations in Broca’s area; importantly, these neural changes were associated with the severity of respiratory impairment. Together, these findings may advance our understanding of how respiratory impairment can shape the cortical networks that are critical for speech production.

Broca’s area is located in the left inferior frontal gyrus. It is one of the most well-recognized brain regions supporting speech production. It is spatially close to the premotor cortex, which mediates motor coordination and is important for speaking. Given that speaking also engages respiration, we hypothesized that cortical sites that govern lung function may functionally and structurally regulate Broca’s area. Our findings provide important evidence to support this hypothesis. Using a simple speaking task, we observed that brain areas such as the left inferior frontal gyrus (BA 44/45; MNI: −51, 9, 27; −45, 39, 21) and precentral cortex (BA 6; MNI: −45, 0, 45), which are essential for speech production, exhibited much weaker activity in COPD patients. Crucially, we extracted literature-based ROIs related to lung function and identified significant differences in activation between HC and COPD participants in regions such as the left pars opercularis (MNI: −54, 8, 16) and left pars triangularis (MNI: −50, 36, 22), which control lung functionality. These findings suggest that lung function may shape Broca’s area as the speech production center.

Lung diseases can affect brain health through multiple mechanisms, including neural pathways, immune regulation, inflammatory responses, and the microbiome, thus forming a bidirectional communication network for the lung–brain axis (Huang et al., 2025). The disruption of neural regeneration may impair these modulatory signals, thereby compromising both lung repair and cortical plasticity. The hippocampus is one of the few adult brain regions that are capable of neurogenesis and supporting plasticity and repair (Chen et al., 2025); this region also showed weaker activation in COPD patients than in HCs, suggesting that pulmonary dysfunction may influence neural regenerative processes and synaptic plasticity.

We also noted that the COPD patients had atypical development in Broca’s area; they had reduced myelination and impaired microstructural integrity measured using *T*_1_. More severe dyspnea was associated with less well-developed microstructure in Broca’s area as well as reduced activation in this region. Although these findings are compelling, the present study was not designed to establish causality between pulmonary dysfunction and alterations in Broca’s area; thus, any causal interpretation should be approached with caution. Future research using longitudinal or mediation designs will be necessary to more reliably test causal links between lung function and neural plasticity in Broca’s area and other language-related regions. Incorporating biomarkers of neural regeneration (e.g., neurotrophic factors or neurogenesis markers) and detailed respiratory measures may help to clarify the mechanistic pathways linking lung function to language network development and maintenance.

Nonetheless, our interpretation aligns with the lung–brain axis hypothesis (Hosang et al., 2022), which suggests that pulmonary conditions can influence brain function. Previous research has focused on how lung microbiota modulates neuroinflammation and autoimmunity. Our findings extend this framework by indicating that chronic respiratory impairment may also affect speech-related regions (particularly, Broca’s area). These insights also suggest that interventions targeting neural regeneration or neurotrophic support may help to mitigate cortical dysfunction associated with chronic lung disease, thereby providing a potential avenue for therapeutic strategies aimed at speech and language impairments.

We observed no significant differences in the baseline-evoked brain activity or cognitive scores between the two participant groups, indicating that our findings were closely related to speech production. Collectively, our results indicate that the lungs may function to shape Broca’s area as the speaking center; this is consistent with the findings of recent research using animal models to investigate the lung–brain axis (Hosang et al., 2022; Park et al., 2024). Our findings suggest that lung function may not only support respiratory physiology but also modulate the neural substrates underlying speech production. This insight offers potential clinical implications for the treatment of speech and language disorders, such as stuttering or speech delay. Additionally, interventions targeting neural regeneration may help to mitigate cortical dysfunction in chronic lung disease, thus offering potential strategies for speech and language improvement. For example, stem cell therapy can regenerate damaged respiratory system structures and restore lung function. Calzetta et al. (2022) conducted a systematic review and meta-analysis of 11 studies involving 371 COPD patients, and reported that stem cell-based regenerative therapies and derived products produced a trend toward improvement in forced expiratory volume in 1 s as well as a significant increase in exercise capacity (measured using the 6-minute walking test). Because efficient speech production critically depends on respiratory support (Chukwu et al., 2025), enhancements in lung and breathing function may indirectly alleviate language impairments. Moreover, respiratory-based interventions can optimize respiratory system support for speech production (Ahmed et al., 2024; Ismailova, 2025), thereby improving speech clarity, fluency rhythm, and sustainability. For example, in a cohort of 343 individuals with stuttering, Ahmed et al. (2024) noted that irregular breathing patterns (e.g., shallow or rapid breathing) were strongly associated with worsened speech disfluency, whereas conscious breathing control and deep breathing alleviated symptoms in a subset of participants. These findings highlight the importance of respiratory regulation for speech fluency and support the potential of interventions targeting lung and breathing function to improve language and speech.

## Limitations

Given that this study included patients with COPD spanning from mild to extremely severe, residual hypoxia or medication use (e.g., corticosteroids) may have influenced brain activity. Removing the effects of these possible confounders will require large, severity-stratified cohorts with detailed treatment histories. Similarly, although increased *T*_1_ values in the COPD group may reflect reduced myelin content, *T*_1_ relaxation time is also influenced by iron and extracellular water contents (Stüber et al., 2014; Vandeleene et al., 2023), indicating that incorporating additional biophysical indices (Mezer et al., 2013) might improve the specificity of microstructural inferences. In addition, all participants in our study were male, which limits the generalizability of our findings. Future research may investigate whether the neural alterations observed in our study can be generalized across sexes. Another limitation of our study was the relatively small sample size and the cross-sectional design, which restricted our ability to establish causal relationships between lung dysfunction and neural alterations. Longitudinal studies with larger samples are needed to confirm the temporal dynamics of lung–brain interactions and to enhance the generalizability of the findings. Finally, incorporating biomarkers of neural regeneration and more comprehensive measures of lung function may also provide deeper mechanistic insights.

## Conclusions

Our results suggest that Broca’s area, which is central to speech production, is modulated by pulmonary function. In COPD patients, dyspnea severity was linked to both reduced activation and altered microstructural integrity in Broca’s area. These findings provide direct evidence that the lung–brain axis may influence the neural basis of speech production. Crucially, pulmonary dysfunction may compromise neural plasticity and regenerative processes in speech-related cortical regions, thereby suggesting a mechanistic link between respiratory health and language function. Our findings also point to potential therapeutic avenues: interventions that target neural regeneration or respiratory support may mitigate cortical dysfunction and improve speech outcomes in patients with chronic lung disease.

## Additional files:

***Additional Table 1:*** *Demographic information of the participants.*

***Additional Table 2:*** *MNI coordinates of the activation peaks.*

***Additional Figure 1:*** *fMRI paradigm and stimuli.*

Additional Figure 1 fMRI paradigm and stimuli.

***Additional Figure 2:*** *Statistical parametric maps of group activation differences between tasks (FDR-corrected P < 0.05 with a spatial extent of n ≥ 10 voxels).*

Additional Figure 2 Statistical parametric maps of group activation differences between tasks (FDR-corrected *P* < 0.05 with a spatial extent of *n* ≥ 10 voxels).

## Data Availability

*All relevant data are within the paper and its Additional files.*
